# Engaging Sub‐Saharan African Migrants in Social and Health Studies in Australia: Research and Ethical Challenges

**DOI:** 10.1002/eahr.60019

**Published:** 2025-07-14

**Authors:** Andre M. N. Renzaho, Michael Polonsky, Julie Green

**Affiliations:** ^1^ Distinguished Professor at the Translational Health Research Institute in the School of Medicine at Western Sydney University and a Bridging Divides Scholar of Excellence at Toronto Metropolitan University; ^2^ Alfred Deakin Professor in the Deakin Business School at Deakin University; ^3^ Adjunct professor in the School of Medicine at Western Sydney University, an honorary principal research fellow at Murdoch Children's Research Institute, and a senior fellow in the Department of Paediatrics at the University of Melbourne

**Keywords:** human research ethics, research with migrants, research with refugees, ethical challenges, migrants, sub‐Saharan Africans, refugees, collectivism

## Abstract

The study summarizes and discusses challenges in engaging Sub‐Saharan African migrants in Australia in social and health studies using data from 15 discrete projects co‐led by the three researchers who authored this article. The projects included cross‐sections of the African community, focusing on parents and their children, and were carried out over 11 years (2007 to 2018) in Australia. An African Review Panel (ARP), a community‐owned steering committee whose members were drawn from the target communities, oversaw the implementation of these projects. Directed content analysis of textual data, drawing on reflective practice through ARP interactive reflective meeting sessions and bilingual workers’ reflective field notes, was undertaken. Findings and associated learnings were summarized into broad themes around lessons learned from participatory research and ethical challenges. Current guiding ethical principles in research may not cater to all cultures, and there is a need to develop ethical guidelines that are culturally responsive to account for collectivist values related to cultural expression and experiences.

Over the last four decades, as a result of severe economic difficulties, increased poverty, political instability, and professional aspirations, the number of African migrants relocating to Organisation for Economic Co‐operation and Development (OECD) countries has increased from 7.2 million in 2001 to 12.5 million in 2016 and 19.5 million in 2020.^1^ African migration to Australia has been driven by different circumstances. For example, early African migration waves were predominantly white South Africans coming on skilled migrant and business visas.^2^ Large‐scale intake of black African migrants did not occur until the turn of the twenty‐first century, mostly through Refugee and Humanitarian Program entry visas and the family reunion stream, with limited migration under the skilled migrant stream. Currently, the number of people of African origin residing in Australia has been estimated at over 496,000, representing roughly 1.7% of the Australian population and 5.1% of Australia's foreign‐born population,^3^ with black Africans from sub‐Saharan African (SSA) countries accounting for 42% of all African‐Australians.^4^ Once in Australia, SSA migrants are faced with many social and health issues that impact integration and social, emotional, and physical well‐being. Migrants from developing to developed countries frequently face difficulties related to language, finding housing and employment, knowing how to use public transport or the banking system, understanding the legal system, or accessing and utilizing health services.^5^

As a result of changing migration patterns, after the year 2000, research has been focused on understanding community needs and health profiles, as well as informing ongoing governmental and nongovernmental support programs. Most SSA humanitarian entrants lived in remote areas and sojourned through under‐resourced refugee camps before coming to Australia under Refugee and Humanitarian Program entry visas and family reunion streams (reunification with separated family members and friends who often remained in refugee camps and war zones).^6^ Perhaps not surprisingly, these entrants have had limited exposure to or experience with participating in or contributing to academic research.^7^ However, they likely experienced population surveys in refugee camps to assist them in securing the documentation they needed to demonstrate their entitlements and to determine their eligibility for humanitarian assistance such as food rations, accommodations, and welfare services.^8^ A camp registration card was given to all heads of families, which entitled every family member to subsistence allowances and various social, financial, and health services. Centering the camp registration card on men as breadwinners and providers for their families conferred on them an important role to play when members of their families had to participate in community surveys.^9^

Participation in survey processes within refugee camps is directly linked to participants receiving services or support from governments, advocacy groups, or other support agencies that do not require institutional ethics approval associated with academic research. As Hugman and colleagues note: “[T]here are many complex ethical challenges that stem from a combination of the precarious situation of refugees themselves combined with a risk that, for some researchers, the ends may justify the means, leading to ethical lapses.”^10^ Additionally, undertaking academic research is essential to better understand how to assist these migrant communities with integration into host societies and economics. However, in host countries, each family member can act autonomously, which may threaten family power structures. Conflicts may arise between the ethical requirements for the conduct of academic research and cultural expectations. The aim of our study was to discuss and summarize the research and ethical challenges of engaging SSA migrants in community‐based social and health research in Australia, taking a reflective practice approach that draws on data from 15 discrete but complementary mixed methods studies undertaken by some members of the research team that authored this paper. By articulating these challenges, we aim to make an important contribution to the policy discourse and research engaging with recent refugee and migrant African communities in shaping the design and ethical considerations that account for the customs and practices of communities participating in research.

## METHODOLOGICAL APPROACHES

A total of 2,258 human participants, 114 bilingual workers, and 48 African Review Panel (ARP) members were involved in trying to enhance practices or performances in various stages of implementation of the 15 projects.^11^ Table 1 (available online; see information

**Our team found that researchers’ beliefs about engaging communities as being a challenge due to language difficulties did not hold if a considered collaborative community engagement effort was undertaken**.

about accessing this material in the “Supporting Information” section at the end of this article) is an overview of the 15 projects used to inform this paper. It provides the study design, the sample size, and research and ethical issues that arose. The projects were carried out over 11 years (2007 to 2018) in the Australian states of Victoria, New South Wales, and South Australia, and predominantly included refugees, where 78% of participants had Refugee and Humanitarian Program entry and family reunion stream visas, and were largely from Eastern, Central, and Western Africa.

The focus of the studies was diverse, varying from acculturation and obesity prevention,^12^ refugees’ understanding of the new food environment, food beliefs, perceived health,^13^ health literacy and health knowledge gap study,^14^ blood donation interventions,^15^ and mental health,^16^ to parenting and intergenerational conflicts related to educational concerns and expectations,^17^ and parenting in the context of changing parenting approaches and family functioning.^18^ For all qualitative studies, participants were recruited through existing community structures such as religious institutions, community associations, and social groups. Data were collected through a combination of semistructured individual interviews and focus group discussions. For quantitative studies, participants were recruited using a snowball sampling approach, as this approach is often recommended for recruiting difficult‐to‐access populations, such as newly arrived migrants experiencing language barriers or low engagement with the service system.^19^ Newly arrived migrants are also frequently relocating in search of more affordable housing.^20^ Our research team attempted to limit potential bias and improve representativeness by focusing the sampling in local government areas with high concentrations of target migrant communities, to ensure inclusiveness and adequate coverage of the target population. Across studies, participants were compensated for their time with a $15‐$25 gift voucher (depending on the length of the study) and for face‐to‐face qualitative studies, and were provided with refreshments during focus group discussions.

We extensively used trained multilingual research assistants who were recruited from within the target communities to assist with data collection. Materials were first developed in English and then translated into target‐community languages. Recruiting multilingual research assistants from target communities ensured that materials and research questions could be delivered in a language relevant to the various communities and could overcome any literacy issues, as well as ensure that our team was collaborating with various communities, rather than just doing research on these communities. Regardless of the study design, bilingual research assistants were trained to record and maintain reflective fieldnotes, which were discussed during team meetings.

Our team broadly applied a reflective practice approach, whereby “knowledge is generated from practice.”^21^ Using a reflective practice approach to research means assessing what has been done and how the complexities of the process can be improved, by being open minded to issues that arise and considering alternative actions. This requires that the individuals who undertake the research be highly engaged in the practice as well as the reflection of the practice.^22^ As an extension of reflective practice, meta‐reflection on practice involved joint reflective feedback reports from 15 meeting sessions with ARP members and 114 reflective fieldnotes^23^ to discuss research challenges and practice among SSA migrants, and to analyze the notes by moving from description to analytical levels of reflection.^24^ The use of meta‐reflection is critical when developing guidelines or improving performance or practices. The reflective fieldnotes allowed the collection of impressions, as well as the description of data collection environmental contexts, behavioral patterns, and nonverbal cues pertinent to the interpretation of audiotaped data that couldn't be adequately captured by the audio recording itself.^25^

To overcome hierarchical structures and power dynamics that could represent a significant barrier to participation, especially among women and young people, fieldnotes involved taking descriptive notes at the time of the interview and/or after field visits.^26^ Fieldnote taking was guided by incorporating three dimensions:^27^

- **Logistics:** Providing the exact description of date, time, and location of the interview.
- **Particulars about the interview environment:** What happened? Who was there? How many people were there? How was the atmosphere? What was going on?
- **Reflective notes:** What caught your attention? How did it make you feel? What surprised you? What did you find interesting? What is your own reaction to the fieldnotes that you wrote?

## DATA ANALYSIS

Our team used a directed content analysis of textual data generated through structured team‐reflexive discussions.^28^ ARP members and bilingual workers were asked to list issues emerging from meeting minutes and fieldnotes respectively. Each team member engaged in reflective group discussion to answer six personal reflexive questions.^29^

1. In what way might your background and/or experience have shaped how you interpreted what you observed during data collection?
2. In what way might what you observed during data collection affect research processes that governed the project?
3. What was one thing you knew or expected about family dynamics and power decision‐making that manifested during data collection?
4. What surprised you about it?
5. What would you say is your take on your participation in the research? What did you hope to get out of it?
6. What were your fears?

Responses to these questions formed the basis of key concepts and predetermined codes. From the preliminary codes, an initial list of coding categories from data was generated by reflective group discussion with participants following four discrete steps:^30^ (1) recognition of patterns; (2) thinking “systems” and “concepts”; (3) having tacit knowledge and background information; and (4) having relevant information. To ensure flexibility and group dynamics, identified categories were modified within the course of discussions as new concepts emerged inductively. The last stage of group analysis involved making sense of the identified themes and their properties.^31^

## RESEARCH GOVERNANCE

To address the need for community engagement to enhance research participation and community ownership, and to oversee project implementation,^32^ an ARP was established. ARP members were drawn from the target communities. The panel acted as a community‐based steering committee and a bridge between the community and the researchers, mobilized the SSA community members, and assisted with the recruitment of multilingual data collectors and individuals who could communicate the importance of community involvement in research. The panel also provided guidance about cultural and ethical issues that may emerge during data collection and assisted researchers in identifying and exploring the implications of the research findings. ARP members independently reviewed all study materials to establish the reliability of contextual factors such as neighborhood of residence, migration status, or language spoken at home.

## ETHICAL OVERSIGHT

While the ARP is important to ensuring that cultural issues are considered, within Australia, all university and medical research also requires review by a human research ethics committee. Therefore, projects summarized in this paper were approved by the following human research ethics committees: the Eastern Health Human Research Ethics Committee (E46/1213), the Western Sydney University Human Research Ethics Committee (H11213 and H10963), the Australian Red Cross Blood Service Human Research Ethics Committee (2014#24), the Australian Catholic University Human Research Ethics Committee Register (No: 201500012R), the Monash University Human Research Ethics Committee (CF14/1443–2014000678), and the Deakin University Human Research Ethics Committee (2015–001, HEAG‐H 89‐08, and HEAG‐H 20/09).

Research activities are governed by institutional policies but are also required to comply with the National Statement on Ethical Conduct in Human Research 2023, which has been developed by the National Health and Medical Research Council of Australia. These guidelines have been integrated into an online protocol document to guide researchers, institutions, and ethics committees in the conduct of responsible and ethically sound research, and to assess the appropriateness of research that has a high risk, with institutions developing a separate process for low‐risk research. However, guidelines must follow the same principles of the National Statement.^33^

Cultural sensitivities are highlighted in several sections of the National Statement, and include being respectful of potential participants and their culture and beliefs (sections 3.1.16 and 3.1.72), as well as Aboriginal and Torres Strait Islander Peoples (chapter 4.7), but there is no specific chapter that outlines ethical considerations for migrant populations (see chapter 4.8).^34^ Therefore, we relied on human research ethics committees to give feedback on the potential cultural and ethical complexities that might arise. Indeed, all human research ethics committees must have at least eight members: (1) a chairperson with suitable experience in research and the ethics review process; (2) two community members who have no paid affiliation with the institution; (3) a nurse, counselor, or allied health professional; (4) someone who undertakes a pastoral care role in a community (i.e., Aboriginal and/or Torres Strait Islander elder, community leader, or minister or religious leader; (5) a qualified lawyer; and (6) two people with research experience relevant to research proposals being considered. While human research ethics committees must have Indigenous consultation for any research involving Aboriginal and Torres Strait Islander Peoples, there is no requirement that such committees include a person from African communities (or any migrant community). When researching migrant subpopulations, as specified previously, the human research ethics committee does expect that researchers consider cultural issues. Given the cultural diversity of African communities, having suitable representation would potentially involve multiple representatives.

## STUDY RESULTS

Our findings are summarized into broad themes around lessons learned from participatory research and ethical challenges. Lessons learned centered on factors facilitating inclusive community engagement with SSA migrant communities, which covered five themes: (1) effective community engagement; (2) building on existing community structures and resources to understand community power dynamics and gatekeepers; (3) the importance of food for effective engagement, with the potential to create an environment that represents a threat to confidentiality; (4) acknowledging that engaging SSA migrants is a time‐consuming process; and (5) the need for flexibility.

### Lesson 1. Effective community engagement

Our team found that researchers’ beliefs about engaging communities as being a challenge due to language difficulties did not hold if a considered collaborative community engagement effort was undertaken. We found that all SSA communities had bilingual people willing to be mobilized and trained to facilitate the integration of SSA communities in social and health research. The process of the initial establishment of these engagement actors (i.e., the ARP and connections with community leaders) took time (approximately six months), but working with the community resulted in the development of trusting and enduring relationships that ensured that communities remained effectively engaged with the research process, relationships that could be reactivated with each project. In some communities (e.g., from the Democratic Republic of Congo, Somalia, Ethiopia, Eritrea, and South Sudan), there was a greater lack of awareness of the research process and limited English language proficiency. The process of inclusive community engagement addressed this by (1) taking an inventory of community leaders within key community structures, through whom our team sought permission to engage their respective communities; (2) gaining community leaders’ support in identifying and recruiting bilingual workers involved in community mobilization and study activities; and (3) attending community events and functions (e.g., places of worship, drumming groups, women's groups) to share information with community at the grassroots level. We found that these kinds of community engagement activities represented a powerful tool for engaging communities and codesigning research programs that addressed communities’ needs.

### Lesson 2. Building on existing community structures and resources to understand community power dynamics and gatekeepers

Three subthemes emerged. The first issue related to intersectionality in community engagement approaches, or how researchers did not know how to apply an intersectional lens when engaging target communities. The ARP included laypeople and ensured that the research team could achieve intersectionality in terms of gender, migration status, and age to overcome critical multidimensional issues that could impede effective engagement with community structures and gatekeepers. We found that SSA migrants’ community structures were culturally and sociodemographically diverse and problems may have been compounded by some limited educational attainment and broad‐based social inequalities complicated by cultural norms. For example, the highly patriarchal SSA communities experienced challenges with broader individual autonomy in Australia. Such inequalities determined who were seen by the participants or communities to be appropriate to participate and whose opinions were considered valid. Employing intersectionality when recruiting ARP members helped us overcome these challenges through training in research processes and creating a culture of empowerment in community mobilization and research. Additionally, not relying solely on gatekeepers helped address issues related to inequitable distribution of power and control within the target communities, as well as that distribution's impact on the research process.

The second issue related to poorly documented and informal governance processes limiting the researchers’ understanding of these existing community structures. We investigated issues within these communities to explore networks of relationships and mediating structures such as places of worship or community‐based organizations, which allowed us to reach out to the community. We strived to be a visible face outside the research context at community events, facilitating trust and respect between the research team, ARP members, bilingual workers, and the communities.

The last issue related to consultation fatigue (when communities get tired of engaging in a repeated series of research without palpable changes observed at the community level, driven by the research findings), triggered by what was perceived as a tokenistic community engagement approach instead of a meaningful and value‐adding approach. The ARP enabled members to be involved in the planning and implementation of the research and emphasized the involvement of cultural insiders, that is, non‐ARP community members who were respected, listened to, and were well integrated in their communities. This community engagement model diminished power imbalances that could affect the research and incorporated cultural norms and beliefs of the communities in the research planning and development. The ARP enabled wider outreach to include the excluded, less vocal, and vulnerable community members, and the smooth navigation of the gatekeeping mechanisms at the community level, allowing gatekeepers to be integrated into our engagement processes with confidence and without creating conflicts among community members.

### Lesson 3. The importance of food for effective engagement

We found that, at a cultural or community event or while interacting with SSA communities, it was important to eat and accept food being offered by the target communities. It was also important to provide food during focus group discussions. Failure to offer or accept food at an event was seen as being akin to rejecting the person offering it, hence creating a sense of mistrust. Having culturally appropriate food and beverages at a social event was part of SSA culture and thus became a cultural expectation. Discussions during the meal could become a catalyst for publicly disclosing personal lived experiences, posing a potential breach of confidentiality. Hence, researchers tended to provide meals after and not before the focus group discussions.

### Lesson 4. Investing time/time‐consuming process

We found that conducting community‐based research required time and resources for building and maintaining community relationships. These community‐building efforts, such as participating in community events and building trust over time, went well beyond the scope of the research. Having the community actively involved throughout the research process, in everything from design, data collection, interpretation of data, and feedback on results, was very time consuming. However, in many ways, time spent in this way was an important investment. The participatory model enabled sociability as it provided opportunities for researchers to truly engage with the community. The researchers, with the support of ARP members, were able to work more closely with the community beyond the research tasks, including opportunities for informal discussions while sharing a meal and listening. This model broke the barriers of any possible conflicts, gained the trust of the community, and helped build sustainable relationships within the community.

### Lesson 5. Need for flexibility

We experienced several practical or logistical challenges that required adaptation and flexibility, such as:

**Flexible notions of time across cultures**. The dimension of time was perceived differently by researchers, community members, and community leaders. Issues around when activities would start were more fluid than might be considered in Western societies. For example, the majority of participants would arrive between 30 minutes to one hour late to meetings and focus group discussions. This required building time buffers into meeting timelines, with more socializing at the beginning of meetings to allow for delays. Such culturally responsive approaches helped the research team to draw out participants’ experiences in multiple steps (e.g., prefocus group discussion briefing and socializing activities, data collection, and postdata collection debriefing), which meant that data collection appointments took much longer than anticipated.

**Working with the bilingual worker as enablers**. Working with bilingual workers was crucial for effective engagement, as they provided cultural knowledge and context to the research and addressed the language barrier between communities and the research team. This collaborative approach ensured synergy between researchers’ conceptual insights and bilingual workers’ cultural knowledge. However, there was sometimes confusion related to the use of bilingual workers. One issue was the loss of detail during interpretation or workers’ biases arising from reflexivity or selective interpreting when dealing with a sensitive topic. We addressed the issue by having multiple bilingual people running focus group discussions: a facilitator, an interpreter, and a note taker. Each took reflective notes, which were compared at the end of the focus group discussions to identify areas of divergence. All these complex interactions, while methodologically robust, took time and had some additional costs.

**Childcare provision to boost participation**. To make it easy for migrant participants to attend the focus group discussions, it was important to address any potential barriers. One barrier that we identified was the availability of appropriate childcare. Providing this enabled a broader cross‐section of participants, who might not normally be able to participate. Thus, the provision of appropriately trained staff and age‐appropriate activities was often necessary. However, providing this service added another dimension: anyone aged 18 years or older who was engaged in child‐related work such as childcare needed the Working With Children Check certificate as mandated by each state in which we carried out our research; they also needed to adhere to the ethical dimensions related to children's safety and protection from harm. In addition, these childcare workers needed to be culturally competent to meet the cultural needs of the target communities.

**Collectivist approaches**. While we sought to keep focus group discussions to 8‐12 people, we found that participants, as part of their collectivist cultural backgrounds, would often bring others or extended family members. These additional people expected to act as participants and be compensated for their attendance. This increased the group size (and costs of groups) but was more challenging when the additional attendants did not fit within the target community and thus their views could distract from the research focus. For example, mothers bringing husbands along to meetings might insert different views on issues around their children, which, while valid, could distract from the focus on the mothers’ experiences. In some cases, the large number of participants required splitting groups into two, but this necessitated having a larger number of facilitators on standby.

### Lesson 6. Ethical considerations in maintaining confidentiality

We highlight three key cultural learnings that gave rise to ethical considerations related to confidentiality when working with the community.

**Issues of consent: gendered power dynamics and cultural norms**. The existing requirements for informed consent prescribed that all study participants had personal autonomy and agency; fully comprehended the purpose, risks, and benefits of the research; and voluntarily participated. However, in some cases, difficulties in obtaining informed consent occurred that were widely associated with premigration trauma and differing cultural hierarchies, levels of research literacies, and educational and sociocultural backgrounds of study participants. For example, we found that, in SSA communities, individuals usually belonged to families, where the concept of family extended beyond the individual's immediate family to include grandparents, aunties and uncles, in‐laws, or cousins. Individuals, especially women and adolescent girls (with the mother's consent), could not in practice make a decision to participate in research independent of the husband's or father's permission, leading to highly interdependent decision‐making processes. Failure to consider other extended family members’ wishes resulted in intergenerational family conflicts. Their collectivist values clashed with the guiding ethical principles of autonomy and privacy.

We inventoried a few critical issues around informed consent from the women who participated. In our study on understanding cultural factors predicting women's perception of body size ideals, we had instances where married women who provided informed consent to participate also had husbands who insisted on accompanying them to the interview. Even after obtaining their husbands’ permission, the women could not be interviewed without the husbands’ presence. These issues were also evident in the studies examining obesity and its risk factors among adolescents (with their mothers’ consent), assessing acculturation and its impact on physical and mental health among Arabic‐speaking migrants. There were instances where fathers would insist that adolescents, especially girls, could not be interviewed without them being present, despite the fact that we had obtained informed consent from the mothers and the adolescents themselves. These instances of culturally endorsed behaviors might have obstructed the ability of women and adolescents (with the mothers’ consent) to freely participate in the research and could lead to conflicts in patriarchal communities. Husbands interpreted women's emancipation as women trying to be equal partners in traditionally male‐dominated communities. Women consenting on their own gave husbands the impression that wives were claiming too much power outside the hierarchical family system and hence could no longer be “controlled.”

For the husband, the wife's consent was invalid without his permission, thereby challenging again the core notion of the ethical principle of autonomy. When a husband gave permission for his wife to participate and accompanied her on interviews, these actions called into question the ethical principles of privacy, anonymity, and confidentiality, which are typically core elements in guidelines and regulations governing research with humans.

Individuals from refugee backgrounds pointed out that, prior to migration, signing a form or being recorded in interviews, no matter the contexts in which these activities occur, was often associated with police persecution, and thus was a source of anxiety. As well, not being able to understand simplified research terminologies and plain language statement forms, with potential study participants reading these forms in a second language, further increased fears and anxiety about signing consent forms. While it was important to ensure the translation of the participant information sheet and consent form into the first language of potential study participants, it was also crucial for facilitators to be patient while dealing with fears and anxieties that might arise from signing the consent form.

To overcome the above challenges, all interviews occurred in community settings away from the home. Even so, women were afraid to participate without their husbands’ knowledge because it could create marital conflicts. In instances where the husband or father accompanied the participant, we decided to find a compromise—the father or husband could be in proximity of the interview while remaining far enough away that they could not hear what was being said. Having ARP members engage with participants and the community prior to research activities helped in building trust while obtaining consent.

**Gossip: passing along or discussing information about others’ lives in public**. During focus group discussions, there were instances where information provided was seen as communal and not private, no matter how sensitive it was. One example was from our study on understanding cultural factors predicting body size ideal. Women wanted to discuss their friends’ husbands’ dissatisfaction with a thin body type among wives (seen as shameful and the inability for the husbands to provide for their wives). Women used their friends’ marital disputes, friends who were also present at the focus group discussion, to validate their own experiences. Participating women had no problem with their own stories and experience being recounted by other people, but our strict focus group discussion guidelines stated that such discussions should be excluded. Another example was from the study on obesity and its risk factors among adolescents in which, during a focus group discussion, women tried to recount their friend's maltreatment of adolescents with thin body sizes. One mother who was at the focus group discussion encouraged women to discuss her adolescent son's story, because she believed the story exemplified major issues in their communities. The issue related to the adolescent boy's negotiating a dual cultural identity, where the boy received compliments at school for being slender and tall, the body size and shape most of his white peers at school desired to have. However, when the boy returned home from school, he would be told by his parents, especially his mother, that “his bottom was flat” and he needed to eat more foods and was sometimes forced to overeat to put on weight. To satisfy both the family and the school environment, the boy would wear shorts under his pants to have a bulky waist and bottom while at home, thus attracting compliments from his parents. Halfway to school, he would stop to remove the shorts from underneath his pants, and as he returned from school, he would stop to put the shorts back under his pants.

For women in focus group discussions, passing along or discussing information about others’ lived experiences in public was seen as culturally acceptable and a shared responsibility as the information was communal, although we viewed it as contrary to ethical principles. However, we also observed that, despite cultural endorsement, gossip among participants in the focus groups who were from the same community made some women less likely to disclose personal information or even participate in focus groups to prevent their lived experiences being shared. We avoided this by bringing together focus groups with a mix of members from different SSA communities.

**Cultural identity as a potential source of conflict**. We found that recruiting people from the same country did not necessarily reflect a unified subcultural identity, since there were multiple subcultures within countries that could lead to conflict, with the potential to breach confidentiality. For example, for Ethiopian communities, the Oromo ethnic group wanted to be identified as Oromo people and not Ethiopians, which may have alienated other Ethiopian communities who did not see them as a separate group. Putting together people who are from the same country but who identify with different cultural identities proved a potential source of conflict. We avoided this by ensuring focus group discussions were organized in the way cultural groups self‐identified, but data were summarized and disseminated according to the country of origin.

### Lesson 7. Harnessing community trust through research dissemination

**Exactly who benefits?** We found that the most common barrier to conducting effective community‐based research among SSA communities was the sense that these communities felt overstudied without receiving significant benefit to their communities. These communities highlighted what they termed “researchers’ selfishness,” where the data were used for the researchers’ benefit and career progression at the expense of the communities’ needs. Communities insisted on being able to own the data from the research due to their cocreation and coauthorship of research findings. They suggested that consultation fatigue was a form of community exploitation. Our research team discovered that creating opportunities to benefit communities beyond research findings was important, and so, for example, all ARP members and community members recruited as multilingual research assistants were trained in the ethical conduct of research, in approaches to research implementation (including data collection techniques and the mapping of data analyses), and in data dissemination strategies. Research team members acted as mentors for these individuals and provided them with job references. This approach created employment opportunities for community members, with 5‐7 members gaining employment each year.

Findings were disseminated back to the communities and then used to assist in the codesign of community‐based interventions. We have described the process of disseminating results back to the community elsewhere.^35^ Although organizing forums through which to disseminate findings was easily done through the ARP, the main challenges to emerge from this dissemination strategy were the tensions between Western science and Indigenous knowledge and culturally ingrained beliefs and values.

**Conflicting perspectives, assumptions, and priorities**. During our research, community members noted that many research institutions were carrying out research among SSA migrants with similar research questions, leading to competition among these institutions to access the community. The competition created conflicts among gatekeepers and community members. Some gatekeepers fought for monopoly control as a point of entry to the community. There were instances where universities were treating communities as commodities that they owned. Researchers incorrectly assumed that community leaders and members would be keener to support the process of data collection as compared to the development of broader relationships. As a direct consequence of the competition between research institutions to access SSA communities, lack of coordination and collaboration led to a duplication of research and to consultation fatigue.

## DISCUSSION

The review of our past research practices identified two key lessons from participatory research and four key lessons related to ethical challenges. Lessons learned from participatory research related to effective community engagement and understanding community structures and gatekeepers. Lessons related to ethical challenges were: using food for effective engagement; investing time in community building; the need for flexibility; and harnessing community trust through research dissemination while maintaining confidentiality (preserving cultural norms and gendered power dynamics). As well, in Australia and other member countries of the OECD, undertaking academic research requires obtaining prior ethics approval from accredited human research ethics committees before research commences. One aspect of the ethics approval process is to ensure that participation is voluntary and that respondents are fully informed of their rights and obligations.

In recognition of their assistance and support, many academic studies involve some limited payment or compensation, whether monetary or in‐kind. Ethics guidelines specify that reimbursement to participants of costs associated with taking part in research or being paid for time involved is appropriate (National Health and Medical Research Council [NHMRC]'s Guideline 2.2.10), provided that payment is not disproportionate to the time involved.^36^ Ethics principles require that any payment does not unduly influence an individual's decision to participate in research.^37^ However, as NHMRC guideline 2.1.11 states: “decisions about payment or reimbursement in kind, whether to participants or their community, should take into account the customs and practices of the community in which the research is to be conducted.”^38^ In our study, husbands’ expectations to attend their wives’ or children's interviews and be compensated was at odds with ethical principles and could be seen as passive inducement since they controlled how family members would engage in research. In addition, the unique features of SSA migrant communities could raise undue expectations and tensions in the community when considering inclusion in research, especially when there was some payment; for example, the idea that people may not be eligible for a study, for various reasons, could be perceived as being discriminatory. The husbands might question why they would participate and not get paid or receive benefits as was the case prior to migration in refugee camps.

Traditional community engagement approaches are designed to meet the needs of the mainstream populations and, in some cases, might explicitly exclude migrants because of perceived language constraints.^39^ For example, most randomized controlled trials in English speaking countries exclude non‐English speaking praticipants.^40^ A systematic review of articles published in three major Australian health care journals found only 2.2% of articles focused on multicultural health issues,^41^ demonstrating a significant underrepresentation in research, even though other research demonstrated that migrants were equally as willing to participate in research as people from mainstream communities.^42^ Current evidence suggests that migrant communities are not approached or effectively engaged in research and community interventions, thus their limited involvement.^43^ The knowledge gap from the lack migrant community health interventions remains a serious challenge. Poor engagement of migrant populations threatens the generalizability of the generated evidence and associated policies and programs. Given that many randomized controlled trials exclude these migrant populations, recommendations or interpretations from systematic reviews of studies is subject to bias. This is especially

**The most common barrier to conducting effective community‐based research among SSA communities was the sense that these communities felt overstudied without receiving significant benefit to their communities**.

problematic given that work based on disadvantaged communities may in fact exclude those being targeted in policy development research. The ARP was an essential tool to assist in effective community‐based engagement. Adopting this model can facilitate the inclusion of marginalized and visible minorities in research, help align community interventions and research with the cultural and social needs of the community, and empower community members to engage in research activities.

The majority of migrant community interventions have predominantly relied on community steering committees with the most vocal and articulate community members.^44^ However, the most vocal and articulate community members may be keen to protect their own personal interests and push personal agendas, rather than promote the needs of their respective communities.^45^ Relying on such community members could result in failure to build on strengths and resources within the community.^46^ In order to facilitate true community involvement in research participation, our approach of using ARP members sought to understand community structures and dynamics in terms of who represented the community, who in the community was excluded, and the extent to which community leaders represented their respective communities.^47^

Another major issue was related to ethical principles and the challenges dealing with migrants who have collectivist orientations. These cultural orientations underscore the fundamental underlying features of the SSA cultural system, which defines how community structures are negotiated and ways community members and interactions govern decision‐making.^48^ For example, gender was an ethical domain that needed to be considered during the recruitment and consent processes and in the collection of data in traditionally patriarchal communities.^49^ Research in SSA and other collectivist communities has highlighted the need to involve husbands of the women participants during the consent‐seeking process.^50^ Failing to do so resulted in either wives refusing to participate or husbands deterring the wives from taking part in the research.^51^ Husbands’ quest to be involved in their wives’ consenting process is a challenge that is difficult to formally manage, as individuals should ideally have autonomy to make research participation decisions on their own. We have never sought to formalize a spousal approval process in our research, as doing so would explicitly enforce cultural norms and expectations that may be appropriate in other cultural contexts and legal systems.^52^ As Ezeome and Marshall^53^ observe, in Nigeria, as is the case in many African countries, gender prejudices are evident in the consenting processes. Gender roles and hierarchy within the family take precedence over individual autonomy. That is, a woman's willingness to consent is under her husband's authority as the head of the family. In the absence of the husband, a woman's willingness or decision to consent may fall to the husband's brother or father, and in some case the woman's brother.^54^ Without the husband's presence, cultural tensions can arise if researchers approach women and invite them to participate in research that might ask some sensitive questions. Individual autonomy, while recognized, is overridden when decisions to participate are made within the extended family system.^55^

We have never formalized a spousal approval process in our research because spousal control behaviors would fall under family violence laws or intimate partner criminal laws in Australia, depending on the state or territory in which the research is being carried out. Seeking a spousal approval process would also be inconsistent with the Australian National Statement on Ethical Conduct in Human Research and cannot be generalized, since not all migrants and ethnic community families follow traditional family roles. It is widely accepted that requiring partner agreement or authorization for an individual to participate in research is in violation of the principles of autonomy of research participants and their right to confidentiality.^56^ This increasing need to strike a balance between carrying out ethically sound research and maintaining culturally respectful practices in culturally diverse communities will remain a challenge in current contemporary societies. Current ethical guidelines do not sufficiently consider the sociocultural contexts of collectivist communities in which hierarchies of relationships are embedded. These guidelines need to consider and acknowledge the existing hierarchical structures of communities, and have respect for and sensitivity to cultural norms that guide the dynamics of communications and address cultural buffering in the research design and implementation. Failure to bridge cultural differences and the insistence of a “one approach fits all” model of ethical conduct of research can potentially cause friction and hinder effective community participation and research implementation.^57^

In the SSA culture “foods communicate history, memory, feelings and social status.”^58^ Among African Americans, research highlights a highly valuable connection of family and community with food.^59^ We share similar experiences of working with SSA communities in Australia regarding the cultural meaning of food. SSA communities considered food an essential element for cultivating and maintaining sustained community engagements and lively interactions during data collection. We brought this approach to our research, and our experience was that it was well received and appreciated, and feedback from participants validated this approach. However, the process of ensuring that the food provided and the setting in which it was provided were culturally appropriate was complex and could be a serious threat to the confidentiality of information about participants that was disclosed in focus group discussions. Thus, based on input from ARP members, we provided food after rather than before the focus group discussions to minimize collective disclosure of private information prior to the group discussions.

Consultation fatigue and the feeling of being a commodity of research were themes that were prevalent in our studies. Our findings mirror similar studies among ethnic minorities. For example, with Indigenous communities, research has a “bad name” and is linked with mistrust, exploitation, and experiences of being treated as “objects of investigation.”^60^ These experiences and perceptions often lead to hesitation to participate in research among such communities.^61^ Participant exploitation and commodification and their impact on data validity have been ethical issues of concern in research for decades.^62^ Confusion can also arise around requirements that researchers provide research findings to the community. This sometimes leads to a misunderstanding that research results will automatically (and quickly) translate into changes in community programs, which may not necessarily happen. Our approach created employment opportunities for ARP members, with 5‐7 multilingual research assistants gaining employment each year.^63^ Findings were also disseminated back to the communities, and then used to assist in the codesign of community‐based interventions.^64^

Community calendars of events and meetings are governed by existing relationships within community structures and hierarchies and prioritize community engagement activities that potentially lead to social change.^65^ They harness community connections and do not necessarily focus on or include research activities such as data collection. However, we found that building trusting relationships with the community required lengthy and ongoing consultations and engagement and using community events and meetings to initiate community partnerships that involve communities in all aspects of the research process. Our research emphasized sharing the results with the community, getting their input on the findings to assist in interpretation and better understanding of results, and identifying priorities for future interventions.^66^ Implementing these activities constituted an additional validation and appreciation of our target communities, which were well received and considered a respectful aspect of participatory research.

## CONCLUSION

Integrating culturally appropriate approaches that are consistent with ethical principles can be challenging for several reasons. First, African migrant communities cover diverse cultural groups, even within one country and ethnic community, and there is not one “authority” for community approval or insights. Second, for African migrants and others from collectivist cultures, the level of conformance to their home cultural values varies and ethically imposed informed consent requirements may not apply to all, or may be inconsistent with Western research expectations of autonomy. Finally, given the diversity within African communities, it may be impossible to have an appropriate representative from each group on human research ethics committees. This is where the use of the ARP is helpful. In addition, researchers need to build in consent processes that are culturally customized and undertake research that allows for cultural variations. The issues in this paper highlight that existing formalized processes need to be adaptive in order to have appropriate oversight. However, it is also necessary to recognize the variations in the way that research is designed to allow compliance with both ethical and cultural values and expectations, and how such values and expectations may impact community engagement and participation that exists, as well as how research is designed to allow compliance with both ethical and cultural values and expectations. Research policies for cultural communities need to prioritize ethical conduct and community engagement approaches that build on the ones providing oversight for research with Aboriginal and Torres Straight Islander Peoples. Such policies may need to take into account some formal and structured consideration of how to engage with migrants from collectivist cultures in which gender roles and hierarchies are omnipresent. Policies for cultural communities can be developed by building on the ones providing oversight for research with Aboriginal and Torres Strait Islander Peoples, thus not reinventing the wheel.

To address the ethical challenges we identified in our studies and inform the way forward, we suggest building on the ARP model as a successful cultural engagement model with SSA migrant communities that is compatible with the sociocultural values of those communities. The ARP was effective in facilitating access to migrant communities for participant recruitment, coanalyzing data and providing research results to the communities. The model has demonstrated the potential to reduce power imbalances and ensure inclusion of marginalized community members in research, and to navigate the gatekeeping system while building relationships. The model also aligns cultural norms and beliefs of the community in research planning and implementation and empowers community members to improve their health. Working closely with community members in research has helped us understand the challenges they face in accessing and participating in health research. Our findings suggest that building sustainable relationships with the community and integrating community knowledge and expertise into mainstream interventions, thereby empowering the community to be equal stakeholders in research design and interventions, is the best approach to addressing migration‐related inequities in research. Our findings suggest that current guiding ethical principles in research may prove difficult to apply to all cultures. Thus, there is a need to develop ethical guidelines that are culturally safe and competent to account for collectivist values related to cultural expression and experiences.

## ACKNOWLEDGMENT

Studies included in this paper were funded by the Ian Potter Foundation, VicHealth, the Australian Research Council, Deakin University Faculty of Medicine's Research Development Grant, the Australian Red Cross Blood Service, Beyondblue, the Movember Foundation, and Western Sydney University's seed funding.

## Supporting information

The table is available in the “Supporting Information” section for the online version of this article and via *Ethics & Human Research*'s “Supporting Information” page: https://www.thehastingscenter.org/supporting-information-ehr/.

Supporting information
