# Supplementary material for: Engaging Sub‐Saharan African Migrants in Social and Health Studies in Australia: Research and Ethical Challenges
Source: Ethics Hum Res. 2025 Jul 14;47(4):2–17. doi: 10.1002/eahr.60019 (PMC12258616; doi:10.1002/eahr.60019)
Supplement: Supplementary file 1 — Supporting information [file EAHR-47-2-s001.pdf]

Engaging Sub-Saharan African Migrants in Social and Health Studies in Australia: *Research and Ethical Challenges*

ANDRE M. N. RENZAHO, MICHAEL POLONSKY, AND JULIE GREEN

ETHICS & HUMAN RESEARCH, JULY-AUGUST 2025

Table 1: Projects Informing This Paper

Study number (year of study)	Aim	Study design, countries of origin, and sample size	Number of bilingual field workers	Sources of funding	Documented lessons
1<br><br>(2007-2012)	To assess the anthropometric status among children aged between 3-12 years and food habits of sub-Saharan African	A multi-pronged cross-sectional quantitative study.<br><br>A cross-sectional study of 337 children aged 3–	12	Ian Potter Foundation<br><br>VicHealth<br><br>Australian Research Council	One parent consented for the child. No assent was sought because the children age meant they had no capacity to provide informed consent, and assent is still to date not recognized in the NHMRC National Statement on Ethical Conduct in Human Research<br><br>Informed consent from parents was acquired, but if the mother consented, this was not sufficient without the father's permission. Once the father gave permission, the consent was not valid in case

	migrants in Australia	12 years from 139 households.			he requested to be there during data collection and his request was denied.<br><br>Oromo wanted to be identified as Oromo and not Ethiopian.<br><br>We gave $15 for the consenting and participating parent and $15 for the child. However, there was an expectation from fathers that their permission warranted a reward to reduce fathers' gatekeeping.
2 ((2008-2010)	To explore how sub-Saharan African migrant communities living in Melbourne,	A qualitative study involving individual interviews and focus group discussions.	2	Deakin University Faculty Research	Investing time and flexibility in organizing focus group discussion<br><br>Oromo wanted an Oromo-only focus group discussion to be referred to as Oromo and not Ethiopian.

	Australia conceptualize and interpret the Australian food system from an intergenerational perspective and how this impacts on their attitudes and beliefs about food in Australia.	Participants were Eritrean, Ethiopian, Somali, and South-Sudanese refugees in Melbourne, Australia<br><br>N = 40<br><br>21 parents (17 women, 4 men) and 19 young people		Development Grant	Provision of childcare<br><br>Intergenerational differences and conflicts in four domains: the abundance of cheap and readily available processed and packaged foods, nutrition messages that are complex to gauge due to poor literacy levels, promotion of a slim body size that contradicts pre-existing cultural values surrounding body size and shapes, and Australian food perceived as being full of harmful chemicals.<br><br>Main issues related to acculturation gap-distress, a state developing from different acculturation pace between parents and children leading parents and their children holding different values and preferences and associated family conflicts and child maladjustment:

					- The emergence of a hierarchical intra-generational and unidirectional transfer of information and knowledge in families (i.e., from grandparents to parents to children), where children are prohibited from imposing their values or knowledge to their parents or grandparents.<br>- Intergenerational conflict on the slimming culture, which was a significant paradox. Sub-Saharan African refugee mothers often invoked the desire for weight gain to increase their attractiveness, marriage prospects and social status despite being fat was seen as an attractive unattractive in Australia. They showed resistance to pressure to conform to the Western 'thin ideal'. In contrast, the youth preferred a 'slim' or 'athletic' body shape.<br><br>We gave $15 for the consenting and participating parent and $15 for the youth and a culturally appropriate meal. However, there

					was an expectation from fathers that their permission warranted a reward to reduce fathers' gatekeeping.
3 (2008-2010)	To assess intergenerational differences in food, physical activity, and body size perceptions among refugees and migrants from the Horn of Africa living in Victoria, Australia	A qualitative study involving individual interviews and focus group discussions.<br><br>Participants were South Sudanese, Somali, and Ethiopian refugees in Melbourne, Australia<br><br>N = 48 parents	3	Deakin University Faculty Research Development Grant.	Provision of childcare<br><br>Intergenerational conflicts in three domains: food and physical activity behaviors, preference of body size and social expectations, and perceived consequences of various body sizes<br><br>Main issues related to acculturation gap-distress, a state developing from differing acculturation pace between parents and children leading parents and their children holding different values and preferences and associated family conflicts and child maladjustment.

		18 parents and 30 young people			• Conflict and confusion between parents and children regarding meals and traditional foods. Parents considered sandwiches to be an insufficient lunch meal for school. Yet most young people preferred sandwiches as more appropriate for school lunch than traditional food, which they considered required refrigeration, reheating, and cutlery, and was too spicy (taking traditional food to school was not "cool")<br>• Take-away food and social status: For parents, pocket money increased children's food purchasing power and reduced parental control of their children's consumption of traditional foods. When children refused refusal of traditional foods, they were offered take-away foods, which were associated with higher social status (which conflicted with children's learning of healthy eating at schools).

					- Preference of Body Size and Social Expectations: For parents, a large body size was perceived to be beautiful and wealthy. Body size and shape linked to marriage for women, with skinny women perceived not be able to attract good husbands and getting only 'leftover men'. Adolescents deviated from the parents' body shape values toward values accepted and endorsed by peers: slim, tall, and elegant.<br>- Passing along or discussing information about others' lives in public<br><br>We gave $15 for the consenting and participating parent and $15 for the youth and a culturally appropriate meal. However, there was an expectation from fathers that their permission warranted a reward to reduce fathers' gatekeeping.

4 (2013-2016)	To examine factors influencing the participation of migrant communities in childhood obesity prevention initiatives in Melbourne, Victoria	A qualitative study involving individual interviews.<br><br>Migrant parents from African, Middle Eastern, Indian, and Vietnamese origins<br><br>48 migrant parents	8	Australian Research Council<br><br>VicHealth	Cultural influences negatively impacting their healthy lifestyle behaviors and cultural, and family-level and community-level barriers impacting their participation in community initiatives.<br><br>- Cannot interview wife without the husband's permission.<br>- Parents wanting schools to provide them with opportunities for volunteering in school canteens and kitchens including training on healthy cooking, which they stated was of greater value than receiving school notices on healthy eating.<br>- Barriers to children's engagement in physical activity parental fear of crime (mainly drug abuse, racism, and neighborhood crime), concerns about traffic injury, the cost of sport classes, and limited transport facilities.

					- Conveying health messages to migrants was a challenge due to the lack of equivalent words when translating technical concepts.<br><br>Participants received a supermarket voucher valued at $25 as a token of appreciation for their participation in the study.
5 (2011-2012)	To explore how non-diabetic sub-Saharan African migrants residing in Melbourne,	A qualitative study involving focus group discussions.<br><br>Participants were Ghanaian, Zimbabwean,	3	Deakin University Faculty Research Development Grant	Provision of childcare<br><br>A sense of entrapment within an industrialized culture and lifestyle<br><br>For Ghanaian and Zimbabwean migrants. Husbands were supportive of mothers and children (with the mother's consent)'s

	construct and interpret type 2 diabetes mellitus(T2DM) and its risk factors	South-Sudanese, and Burundian migrants.<br><br>N = 61 from 7 focus group discussion			participation in research and were less restrictive in decision-making. For South-Sudanese and Burundian migrants, more husbands' restrictions and less mothers and children's agency in decision-making.<br><br>Passing along or discussing information about others' lives in public<br><br>Participants were given a $15 gift voucher in appreciation for their time and a culturally appropriate meal.
6 (2009-2013)	To examine migration- and socioeconomic-related	A multi-pronged cross-sectional quantitative study	4	Victorian Health Promotion Foundation	Husbands' questioning wives' consents, needing to input into the consenting process.

	influences on obesity among African migrant adolescents in Melbourne, Australia.	Migrants from all Sub-Sharan African regions<br><br>N = 199<br><br>99 parents and 100 offspring aged 13-17 years)		ARC funded project	Husbands' instance on being heads of the family and needing to give wives permission to participate.<br><br>Fathers wanting to be present during interviews with wives and youth.<br><br>We gave $15 for the consenting and participating parent and $15 for the youth. However, there was an expectation from fathers that their permission warranted a reward to reduce fathers' gatekeeping.

7<br><br>(2010-2013)	To document the parenting styles among African migrants<br><br>now living in Melbourne, Victoria, Australia, and assessed how intergenerational issues related to parenting in a new culture impact on	A qualitative study involving focus group discussions.<br><br>South-Sudanese, Somali and Ethiopian migrant families<br><br>N = 85<br><br>A total of 10 focus group discussions (five	4	VicHealth	Provision of childcare<br><br>Accommodating husbands' needs. Mixed gender focus group discussions were included. Investing time and flexibility in organizing focus group discussion<br><br>Mixed-gender focus group discussions did not present a risk around the level of disclosure and comfort among female participants.<br><br>The importance of instilling discipline through corporal punishments and establishing expectations of their children. Fathers wanting to be the focus group discussions with adolescents

	family functioning and the modification of lifestyles	with parents and five with 13-17-year-old children)			despite the mothers being there; fathers' fear of the threat that parental violation of state laws would lead to family separation.<br><br>Main issues related to acculturation gap-distress, a state developing from differing acculturation pace between parents and children leading to family conflicts and child maladjustment.<br><br>Close scrutiny of young people's behavior and social environment: Teenagers are subjected to close supervision in terms of who they visit; with whom they socialize, play, or spend time; the food they eat; and activities they undertake.<br>Young people had to do as they were told by parents without being given an opportunity to provide their perspectives.<br>Parents insisted on not providing choices to their young people, while at the same time seeking to become involved in

					their children's private lives, to assume control of their young people's activities, and to ensure parental demands are met.<br>- Money a source of conflicts: Father wanted to control young people's money.<br>- Passing along or discussing information about others' lives in public<br>We gave $25 for the consenting and participating parent and $25 for the youth and a culturally appropriate meal.
8<br>(2010-2013)	To develop obesity prevention program among Sudanese families in Australia	A qualitative study involving workshops.<br>South Sudanese migrants	4	VicHealth	Provision of childcare<br>The importance of traditional cultural values and intergenerational issues related to health, weight, food, physical activity, and family structure.

		N = 49<br><br>4 workshops, 2 groups 12- to 17-year-old adolescents (n = 32) and 2 groups with parents (n = 17).			Husbands wanting to be at and observe workshops with women.<br><br>Questioning how the program will benefit the community (rollout) beyond church leaders and key community leaders.<br><br>We gave $20 for the consenting and participating parent and $20 for the youth and a culturally appropriate meal.
9 (2009-2014)	To pilot a culturally-competent obesity prevention program for sub-Saharan	Multipronged interactive community-partnered participatory approach that included workshops.	12	Australian Research Council	Provision of childcare<br><br>Main issues related to acculturation gap-distress, a state developing from differing acculturation pace between parents and children leading to family conflicts and child maladjustment:

	African families with children aged 12–17 years.	Participants from various sub-Saharan countries to prioritize health behaviors.<br><br>208 participants (104 parents and 104 offspring).			- The parents' plan included improving parents' knowledge about healthy food options and increasing healthy eating. The strategies for implementation differed dramatically between the parents and children, even when objectives were shared, reflecting intergenerational and age differences. Fathers wanted the final say in children' s decision making processes.<br>- Specifically, the parent strategy for increasing physical activity was role modelling, whereas for adolescents, strategies revolved around organized sporting activities, groups, and competitions with peers (camping, bike riding, basketball), and building self-esteem and self-confidence through fun and easily organized physical activity (dance groups, supportive teachers). Participating in these activities required parental

					control, and the fathers' permission regardless of the mothers' views.<br>- Fathers developed a strategy around preparing food; however, their strategy involved men learning to cook meals, for when the wives were not at home.<br><br>Wanting funding to impellent the program within the community structures, requesting training community leaders for scaling up<br><br>We gave $20 for the consenting and participating parent and $20 for the youth and a culturally appropriate meal. However, there was an expectation from fathers that their permission warranted a reward to reduce fathers' gatekeeping.

10 (2009-2011)	To understand barriers to blood donations by sub-Saharan African migrants	Qualitative focus group discussion<br><br>Migrants from Sudan, Eritrea, Ghana, Congo, and Burundi<br><br>88 participants (43 men; 45 women) from 9 focus group discussions	6	Australian Red Cross Blood Service	Accommodating husbands' needs. Mixed gender focus group discussions were included. Investing time and flexibility in organizing focus group discussion<br><br>Mixed-gender focus group discussions did not present a risk around the level of disclosure and comfort among female participants.<br><br>In fact, single and mixed-gender focus group discussions yield comparable and robust data as blood donation was associated with strong traditional beliefs and values.

					Altruism and anonymity not concepts endorsed by cultural values and blood seen as a symbolic fluid imbued with tribal and kinship properties.<br><br>Giving blood Donating blood to help "strangers" is not viewed positively. Blood is a gift to be given to family members and not strangers.<br><br>Blood equated to health, physical strength, and vitality; hence giving blood was believed to reduce strength and virility.<br><br>Younger people's blood perceived as "stronger" than blood from older people.

					Women who had lost a lot of blood through childbirth were seen as weaker and therefore unable to donate.<br><br>There was an order in which donation seemed to apply. Men and young people donate first, then women, and then older people. Because of this perceived hierarchy, none of the women in the study had donated blood in Africa or Australia<br><br>Participants were given a $15 gift voucher and were also offered a culturally appropriate meal
11 (2009-2012)	To examine whether the theory of planned behavior predict	A cross-sectional quantitative survey	24	The Australian Red Cross Blood Service	Hierarchical gendered collective agency for blood donation behaviors. Wives cannot consent to blood donation without husbands' permission or adhere to traditionally endorsed blood donation order.

	donation intentions among sub-Saharan African migrant communities in Australia and to test the incorporation of blood donation knowledge as an antecedent in the model	Migrants from all Sub-Sharan African regions<br><br>N = 425			Individuals were provided with a $15 gift voucher for participating.
12<br><br>(2012-2014)	To identify the help-seeking barriers and	A qualitative study involving individual	3	Beyondblue and the	Accommodating husbands’ needs. Mixed gender focus group discussions were included. Investing time and flexibility in organizing focus group discussion.

	facilitators for anxiety, depression and alcohol and drug use problems in young people from recently established sub-Saharan African migrant communities	interviews and focus group discussions.<br><br>28 individual interviews and 41 participants from 4 focus groups		Movember Foundation	Mixed-gender focus group discussions did not present a risk around the level of disclosure and comfort among female participants.<br><br>Single and mixed-gender focus group discussions yield comparable and robust data as mental health was associated with strong traditional beliefs and values. No correct translation of mental health in African languages, no sematic equivalence.<br><br>Some words not culturally acceptable (e.g., depression), not recognized as a disease, they think they think they're not sick - the reason why they don't go to specialists.

					Each study participant received a $25 supermarket voucher as reimbursement for their time, and a culturally appropriate meal
13 (2012-2016)	To pilot and evaluate a culturally appropriate health promotion program to promote blood donation within the Australian-African community.	A mixed methods post intervention design.<br><br>Promotional methods were employed including posters, a video and a booklet.<br><br>Target languages: English, Arabic, Swahili, and Kirundi	12	Australia Research Council Linkage Grant	Equal participation of men and women, no restrictions with consents when recruiting women<br><br>Each participant received $30 gift card for their time and travel costs and a culturally appropriate meal.

		7 formative focus groups with 62 participants were conducted to ensure the materials were effective, credible, and culturally acceptable to the target audience, including preferred messages, taglines, and images.<br><br>Survey of 454 sub-Saharan migrants were interviewed in the evaluation process post-intervention			

14<br><br>(2009-2011)	To explore cultural values, practices and behaviours relating to the family dynamic	A qualitative study involving focus group discussions.<br><br>South-Sudanese migrants<br><br>N = 64 from 7 focus group discussions	3	Deakin University Faculty Research Development Grant	Family unity as a core value, a cultural framework through which decisions and the role of family members are determined, and a reference for support and negotiating post-migration experiences and challenges (e.g., The hierarchy of decision making)<br><br>Parenting and youth freedom: parental perceptions of limited control of their children. Parents becoming anxious about children's independence, leading to close control of children activities, such as monitoring phone calls and asking intrusive questions. Parental difficulties with teenagers, especially fathers, due to independence and more rights for children.<br><br>Parents' struggle to preserve cultural values as a mechanism for keeping cultural identity and enforcing controlling traditional practices.

					Changes in gender roles post-migration: A noticeable difference in the role men assume in the family unit such as losing responsibility and power within the family; being expected to be involved in family activities such as cooking, domestic duties or children's school activities; and taking on domestic duties portrayed a sense of failure and cultural humiliation as their decision making at the family level was diminished as mothers took on more responsibilities such as engaging in paid work and decisions that matter to the family<br><br>Each participant received $15 gift card for their time and travel costs and a culturally appropriate meal.

15 (2016-2018)	To explore how migrant youth cope with acculturative stress and intergenerational conflicts, and to better understand the systemic and family-related factors that facilitate positive settlement	A qualitative study involving focus group discussions.<br><br>Migrants from African, Burmese, Nepalese, Indian,<br><br>Afghani, Bangladeshi and Iraqi backgrounds<br><br>N = 164 from 14 focus group discussions,	14	Western Sydney University's seed funding	Mixed gender focus group discussions were included. Mixed-gender focus group discussions did not present a risk around the level of disclosure and comfort among female participants.<br><br>Single and mixed-gender focus group discussions yield comparable and robust data as mental health was associated with strong traditional beliefs and values.<br><br>Family capital as the social solidarity, influence, and control at the family level that governs obligations and expectations, intergenerational knowledge transmission and information flow, social norms, and cultural identity.

	experiences for migrant youth.				Children 'no longer like to associate with their family' and 'prefer to separate from the rest of family members'. Not wanting to meet their extended family, and do not respect or obey their parents; rather, they prefer to stay in their rooms citing 'space', and 'privacy' as reasons for keeping to themselves.<br><br>Parental fear of children disconnecting from the traditional culture and family values led to parents, especially fathers, implement rigid family rules to reinforce traditional values.<br><br>Different restrictions for sons and daughters, boys given freedom in decision making, girls restricted and controlled.

					Each participant received $25 gift card for their time and travel costs and a culturally appropriate meal.
